# Supplementary material for: Intellectual synthesis in mentorship determines success in academic careers
Source: Nat Commun. 2018 Nov 27;9:4840. doi: 10.1038/s41467-018-07034-y (PMC6258699; doi:10.1038/s41467-018-07034-y)
Supplement: Supplementary file 1 — Supplementary Information [file 41467_2018_7034_MOESM1_ESM.pdf]

Supplementary Information

Intellectual Synthesis in Mentorship Determines Success in Academic Careers

Jean F. Liénard[1,2*], Titipat Achakulvisut [3], Daniel E. Acuna [4] and Stephen V. David[1]

[1] Oregon Hearing Research Center, Oregon Health & Science University, Portland, Oregon 97239-3098, United States of America

[2] Okinawa Institute for Science and Technology, Onna-son, Okinawa 904-0412, Japan

[3] Department of Bioengineering, University of Pennsylvania, Philadelphia, Pennsylvania 19104, United States of America

[4] School of Information Studies, Syracuse University, Syracuse, New York 13244, United States of America

Corresponding author: * jean.f.lienard@gmail.com

Supplementary Figures

Interaction between mentor proliferation rate and publication similarity

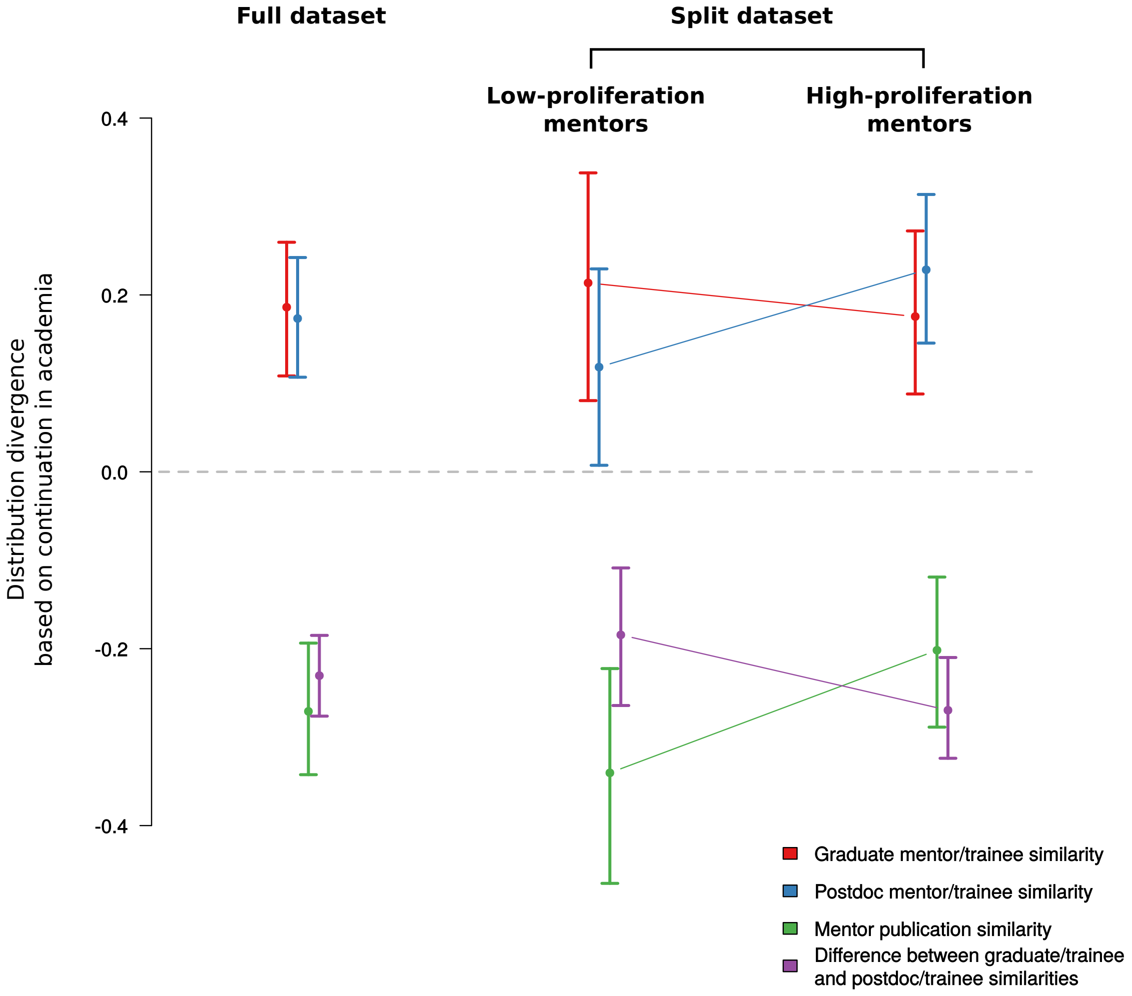

Supplementary Figure 1: Difference in publication similarity between the group of trainees who also mentored at least one trainee in turn, and the group of trainees without mentoring reported in the dataset. The metrics shown are the same as in Fig. 3 in main text, points represent the average difference and the error bars the 95% confidence intervals. "Full dataset" refers here to all mentors. "Lower half" and "Upper half" show these differences when computed only on the low-proliferation and high-proliferation mentors (split based on the highest proliferation rate of the pair). Overall, we see similar patterns as the ones shown in Fig. 3, i.e. a beneficial pattern of higher similarity of trainees with individual mentors on continuation (red and blue traces), and detrimental patterns associated with mentor publication similarity (green) and higher proximity with graduate advisor compared to postdoctoral advisor (purple). There is no clear connection between the publication similarity patterns and mentor proliferation rates.

Influence of graduate mentor only

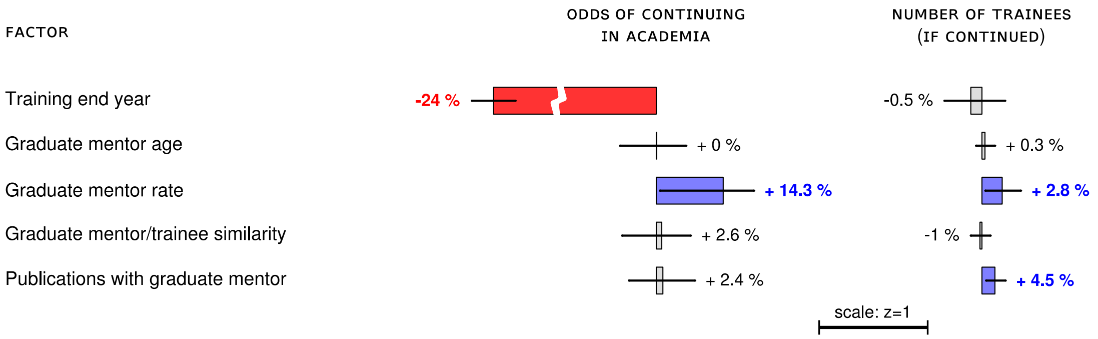

Supplementary Figure 2: Coefficients of a model focused on graduate mentor features (academic age, training rate, co-publishing and similarity with trainee) and excluding features related to postdoctoral studies. This figure shows the regression coefficients computed using only features from graduate studies. We find similar coefficients in these partial regressions, showing that the effects of graduate mentor features are independent from the effects of postdoc mentor features. Of interest, graduate mentor age is still non-significant in this analysis, showing that a possible impact of graduate mentor age is not masked by correlated features from the postdoctoral mentor. This additional analysis was computed on the same triplet dataset used in the rest of this study, and it does not include graduate mentor / trainee dyads without a postdoctoral mentor. There may be different effects of graduate mentors in this dyad group, as graduate students can move directly to independent positions or choose to discontinue training before pursuing a postdoctoral fellowship.

Alternative models of postdoctoral training effects

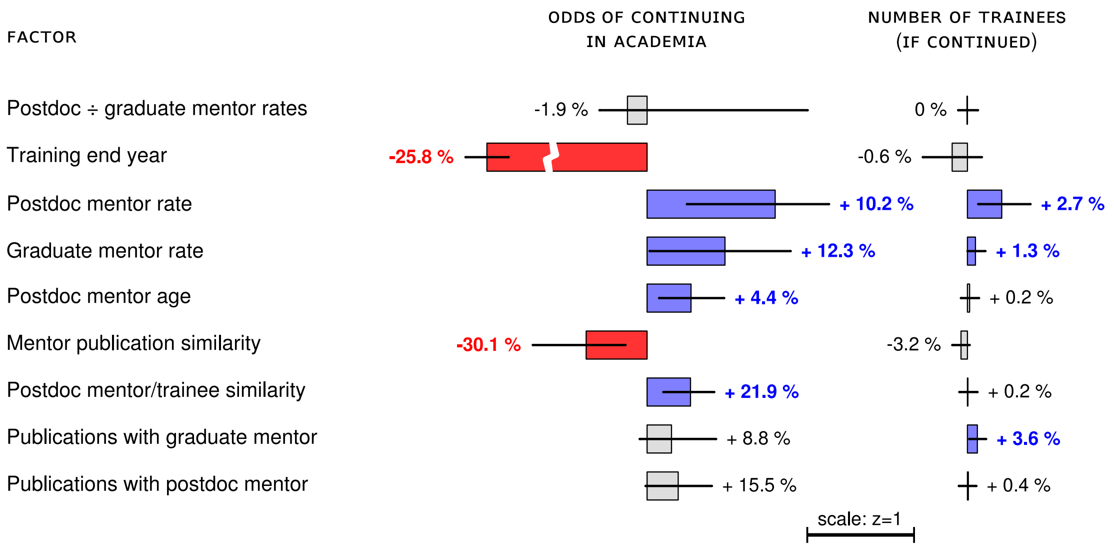

Supplementary Figure 3: Alternative model computed with an additional interaction term, "Postdoc ÷ graduate mentor rates", which was designed to be high for trainees that moved to a postdoctoral mentor with higher proliferation rate ("upward mobility" hypothesis). The lack of significance for this additional term showed that there is no systematic benefit associated with such a strategy.

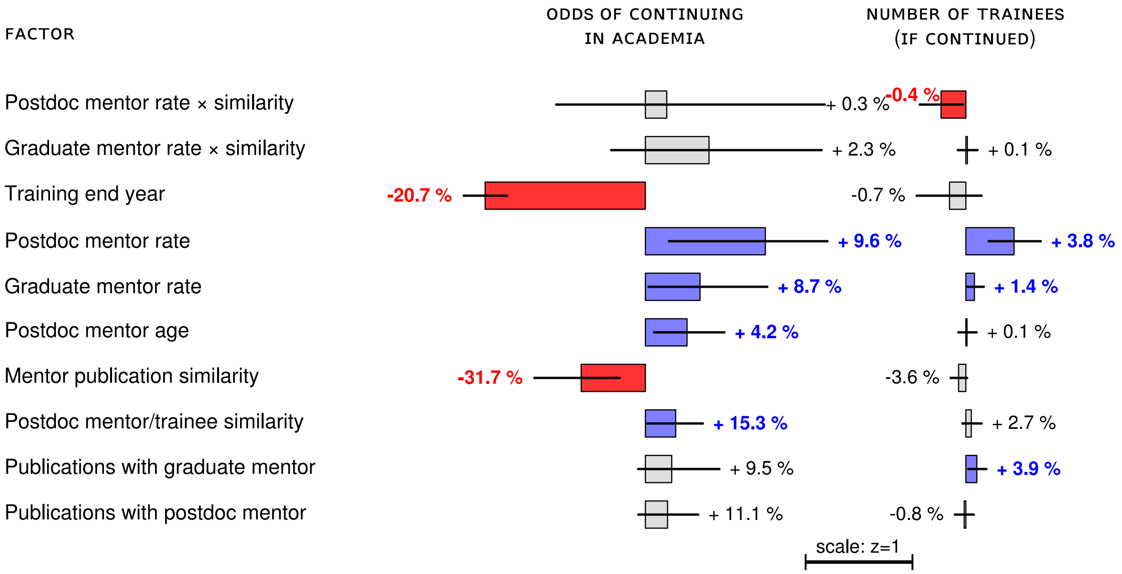

Supplementary Figure 4: Alternative model computed with a new interaction term, "Postdoc mentor rate × similarity", contrasted with a second term: "Graduate mentor rate × similarity". The first term should be high for trainees who moved into the "better" subfield of their postdoc mentor, while the second term contrasts this effect and should be high for trainees who stayed in the "better" subfield of their graduate studies. These terms were not linked to increased (or decreased) odds of finding a permanent position in this dataset. Interestingly, there is a slight influence of the "Postdoc mentor rate × similarity" term on long-term proliferation. One interpretation is that it corresponds to a long-term fatigue effect of disengagement from opportunistic trainees who embraced the research line of their postdoctoral mentor. However, it may also be a spurious effect that parallels the increased long-term effect of the "Postdoc mentor rate" in this regression, compared to the original regression.

Time-dependence of regression coefficients

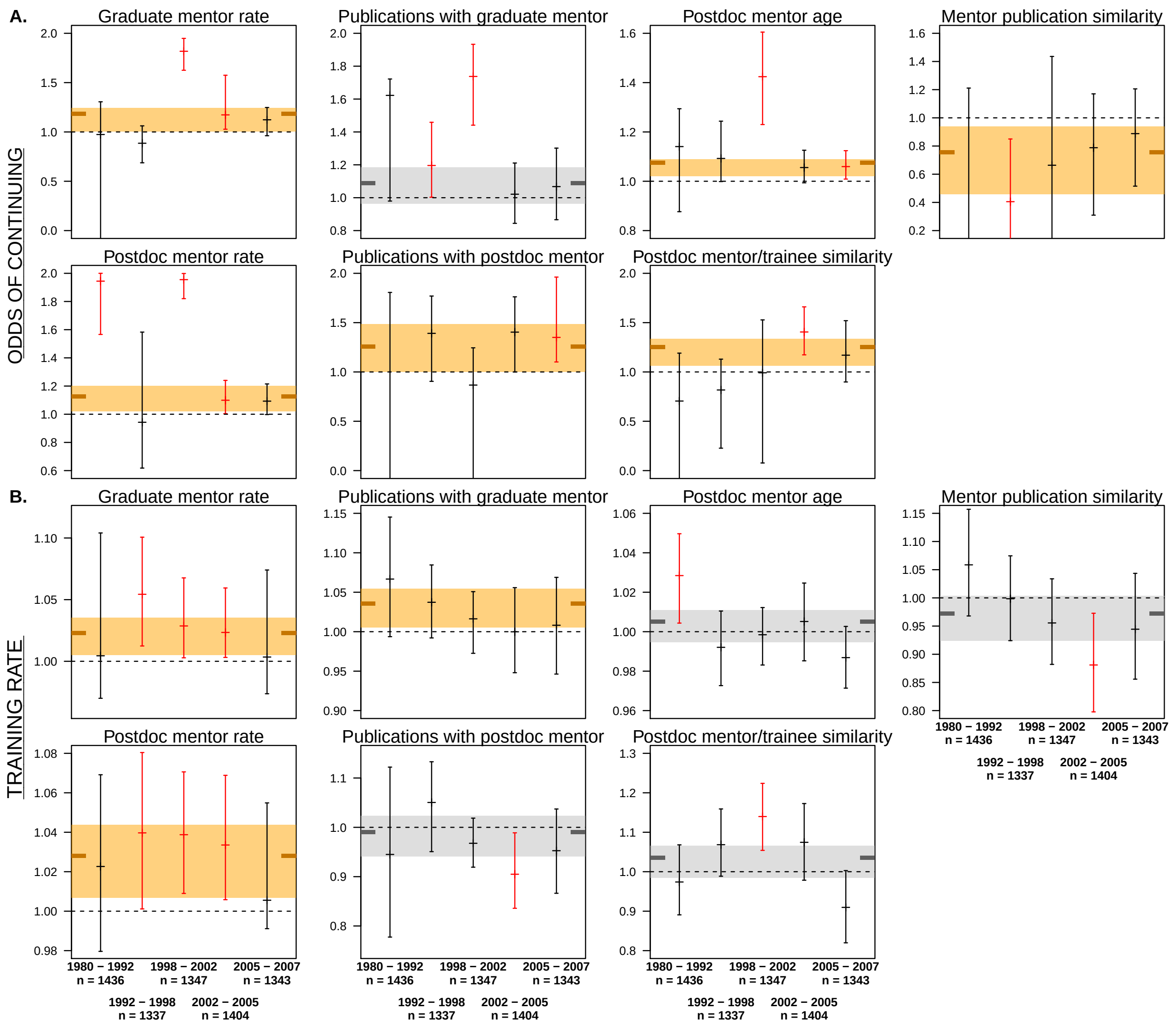

Supplementary Figure 5: Regression coefficients obtained when optimizing the model on different temporal subsets while removing the postdoc end date from the set of predictors. Temporal partitions were chosen to contain roughly equal sample sizes, and were as follows: 1980 – 1992 ($n = 1,436$); 1992 – 1998 ($n = 1,337$); 1998 – 2002 ($n = 1,347$); 2002 – 2005 ($n = 1,404$); 2005 – 2007 ($n = 1,343$). The coefficients estimated to predict continuation in academia (A) and long-term mentoring rate (B) are consistent with the ones obtained with the full model that includes temporal predictors (represented in these plots as shaded areas). Specifically, whenever a variable reaches significance within a temporal subset, it does so in the same direction as the trend apparent in the full model. Reciprocally, whenever a variable reaches significance in the full model, there is at least one temporal subset when it reaches significance as well.

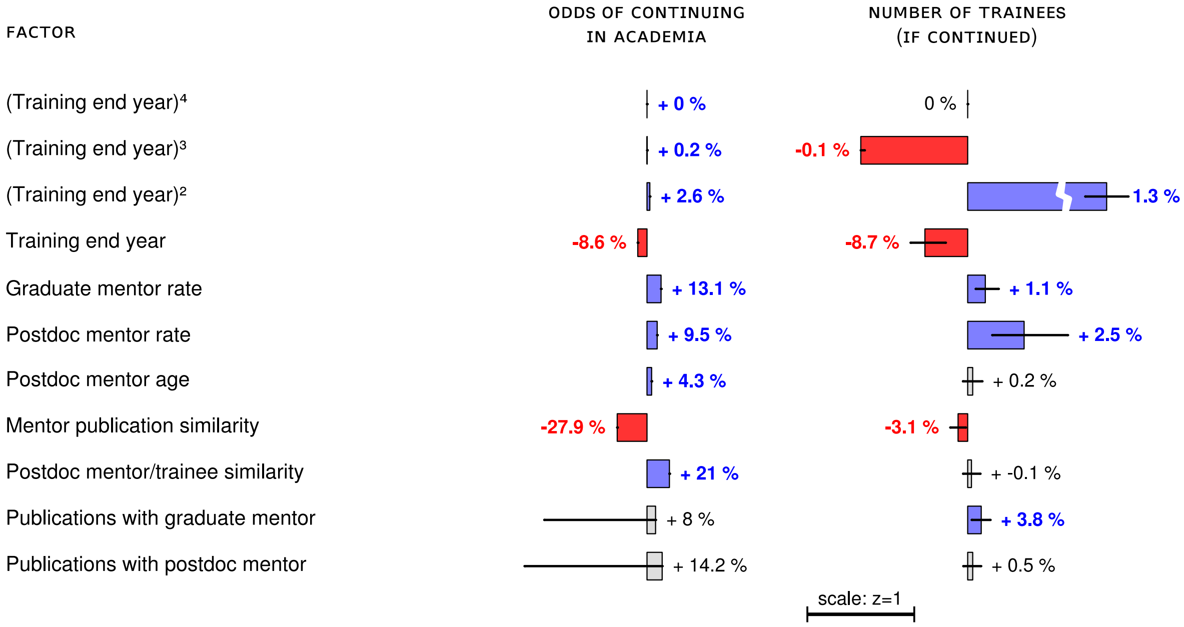

Supplementary Figure 6: Model coefficients, similar to Fig. 4 in main text but with the inclusion of more complex temporal trends, captured here through a series of higher order polynomial terms: (Training end year)^2, (Training end year)^3 and (Training end year)^4. This controls for long term temporal effects through a series of higher-order terms into the regression model. This method has the advantage of modeling arbitrary temporal trends, as in Taylor series, to the risk of over-fitting temporal trends[1]. Not surprisingly, these additional terms were able to capture some additional variance in the data, but they had no large impact on the other factors of interest in the regression. In particular, the intellectual synthesis effects appeared robust to the inclusions of finer temporal controls.

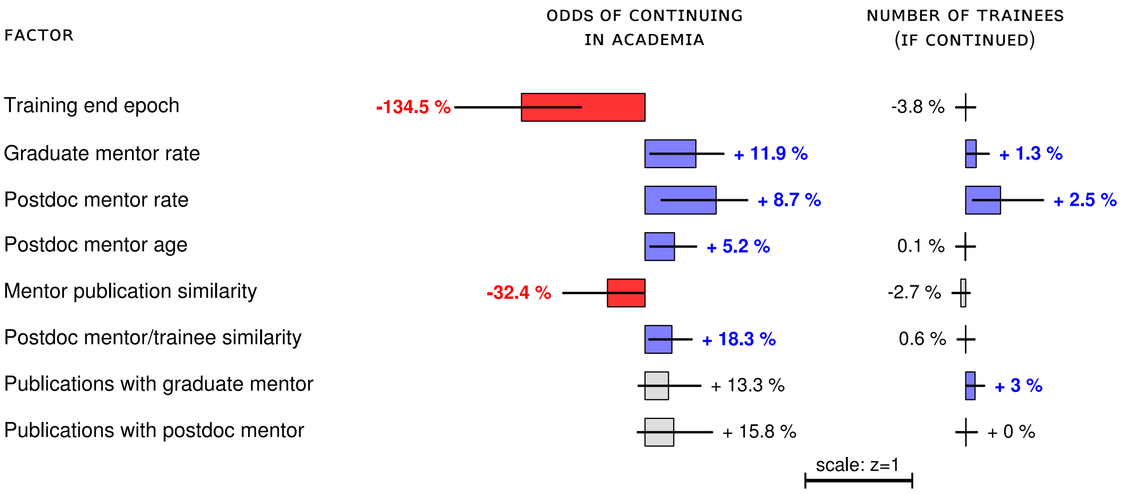

Supplementary Figure 7: Model coefficients, similar to Fig. 4 in main text but with a simpler temporal modeling that uses the ordinal variable "training end epoch". This alternative model for nonlinear temporal effects replaces the continuous time variable ("training end year") with an ordinal variable capturing the temporal subdivisions (numbered from 1 to 5). This transformation did not affect which coefficients were significant, nor did it change the order of magnitude of their effects.

Consistency of effects across fields

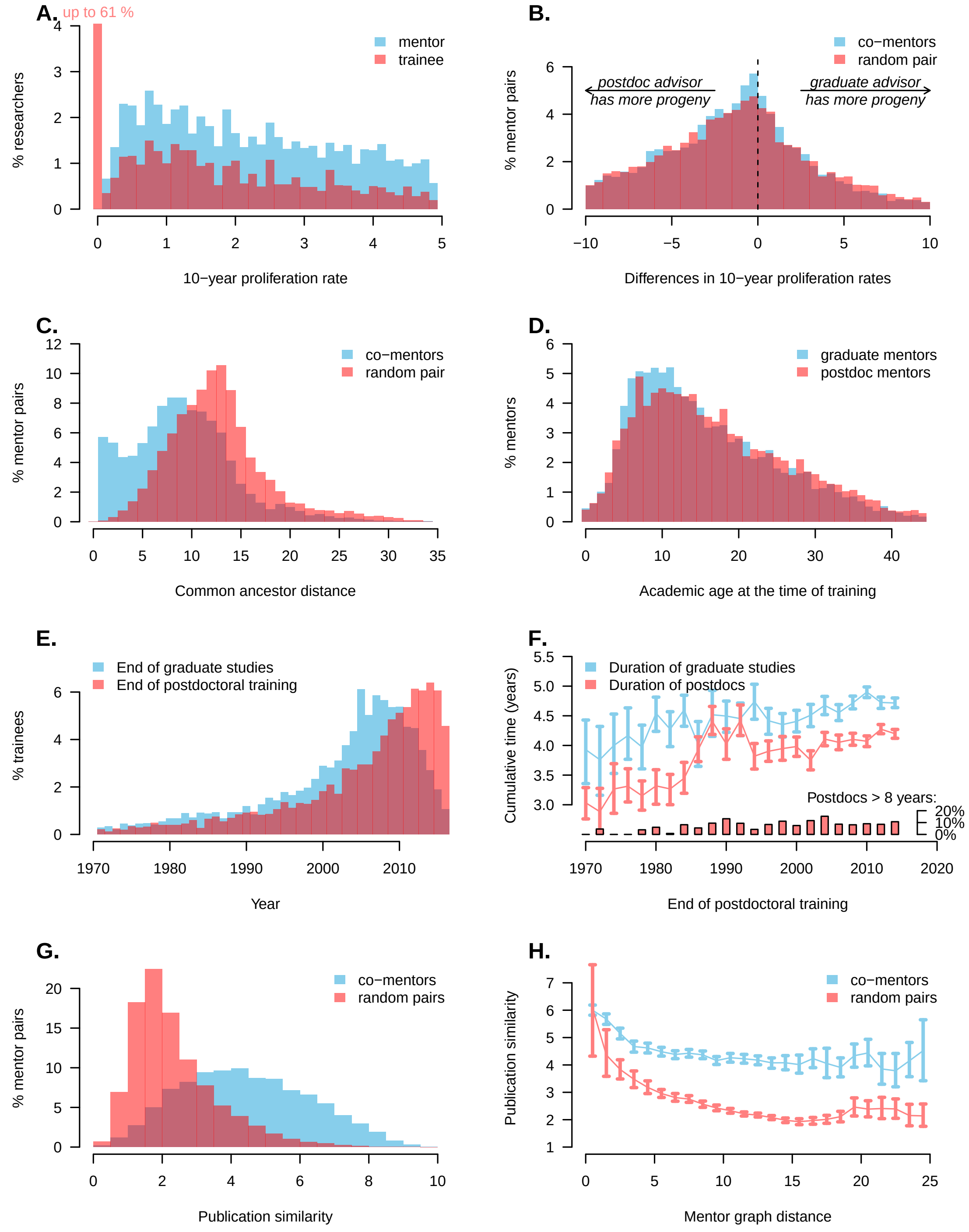

Supplementary Figure 8: Main features of mentorship triplets, plotted as in Fig. 2 in the main text, but using only data for neuroscience graduates ($n = 14,953$ triplets).

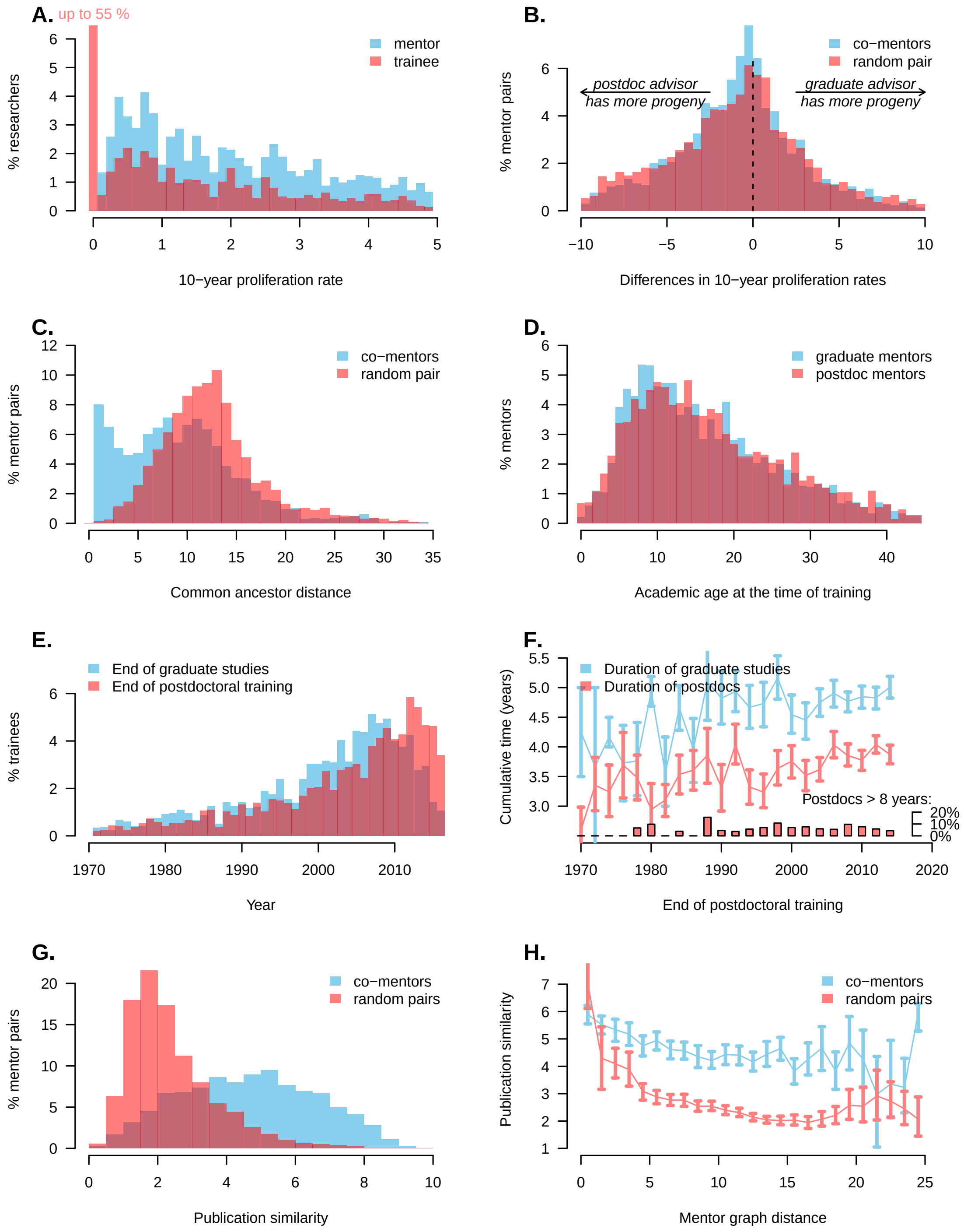

Supplementary Figure 9: Main features of mentorship triplets, plotted as in Fig. 2 in the main text, but using the general population of life science trainees (complementary set of the one analyzed in Supplementary Fig. 8). This includes multi-disciplinary life science researchers, as long as at least one reported field is not neuroscience ($n = 5,742$ triplets).

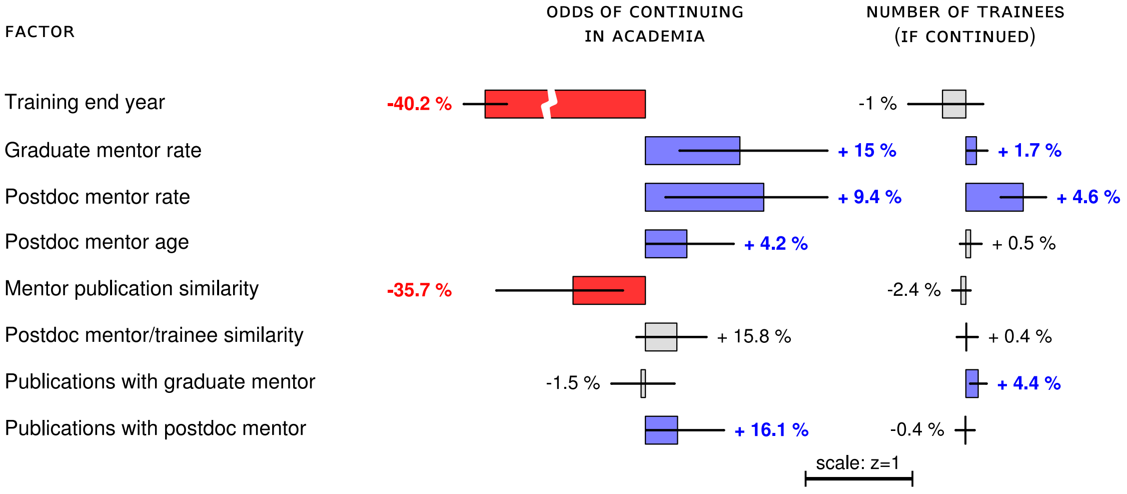

Supplementary Figure 10: Same as Fig. 4 in main text, but using data of neuroscience graduates (corresponding to Supplementary Fig. 8).

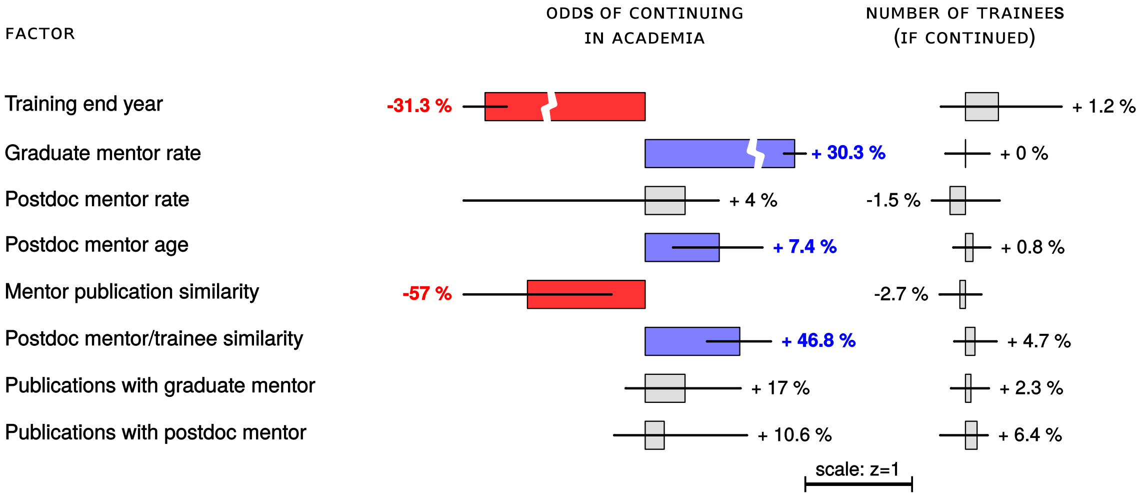

Supplementary Figure 11: Same as Fig. 4 in main text, but using data from general life science graduates, including multi-disciplinary researchers (corresponding to Supplementary Fig. 9). Note that as this dataset also includes multi-disciplinary researchers belonging to different fields including neuroscience, we gave a half weight to this data in the regression to allow comparison with the "neuroscience only" dataset in Supplementary Fig. 10.

Cross-validated model predictions and data

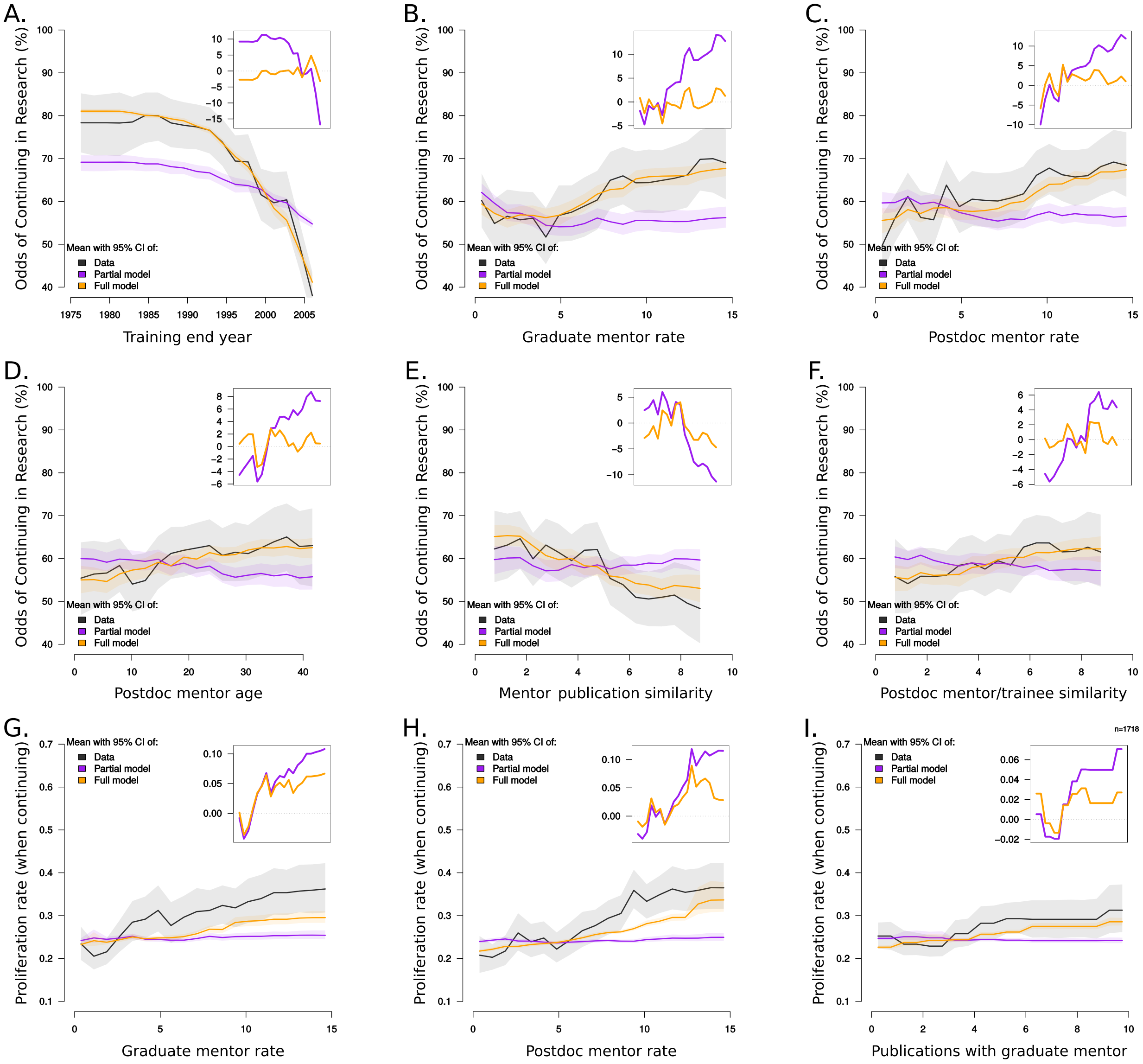

Supplementary Figure 12: Detailed prediction analysis for probability of obtaining an independent research position (A - F) and proliferation rate (G - I), explained by the most significant variables. For each variable, data is shown in black, and cross-validated prediction of the model, including this variable, is shown in orange ("full model"). To visualize the contribution of each variable, we also display cross-validated predictions of the model without this variable in purple ("partial model"). Lines and shaded areas represent respectively the mean values and their 95% bootstrapped confidence interval.

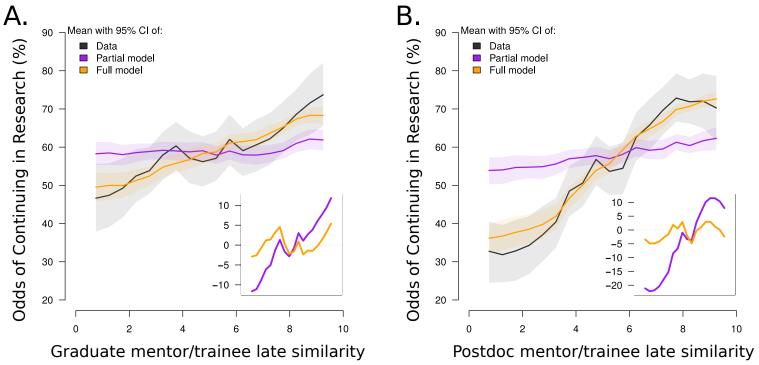

Supplementary Figure 13: Contribution of post-training publication similarity variables to the odds of continuing an academic career, for graduate (A) and postdoctoral mentors (B), plotted as in supplemental Fig. 12. Mentor/trainee similarity displays a stronger effect when publication data is included from after the end of training (compared to using only data available at the end of the postdoc, cf. supplemental Fig. 12F). Also, similarity to the postdoctoral mentor continues to have a larger influence than that of the graduate advisor.

Supplementary Tables

Availability of date information

Availability of date information	
Start of PhD	51%
End of PhD	66%
Earliest publication date with graduate advisor	74%
At least one graduate date available	90%
Start of Postdoc	57%
End of Postdoc	39%
Earliest publication date with graduate advisor	71%
At least one postdoc date available	89%

Supplementary Table 1: Statistics of data availability based on publications and manually entered dates

Model and variable selection

	$\Delta\mathscr{L}$ in cross-validation			
	HP	ZIP	HNB	ZINB
temporal	−10.24	−10.23	−0.47	−0.45
network	−9.45	−9.40	−0.54	−0.30
publications	−10.42	−10.38	−0.71	−0.52
temporal + network	−9.12	−9.11	−0.07	−0.05
temporal + publications	−10.10	−10.09	−0.39	−0.38
network + publications	−9.31	−9.26	−0.47	−0.24
temporal + network + publications	−8.97	−8.96	−0.03	**0.00**

Supplementary Table 2: Impact of model architecture and parameter type on prediction accuracy. The models compared are: hurdle Poisson (HP), hurdle negative binomial (HNB), zero-inflated Poisson (ZIP) and zero-inflated negative binomial (ZINB). Predictors were grouped into the same categories as in Table 1 in main text: "temporal" for the year of the end of the postdoctoral appointment as well as the total duration of postdoctoral training; "network" for the proliferation rate of mentors, their academic age at training and their common ancestor distance; and "publications" for the semantic similarity between mentors' publications (prior to meeting the trainee) and between those of the postdoctoral trainee and mentor (prior to training). The values displayed are cross-validated log-likelihoods aligned on the best model ("ZINB" model using "network + publications + temporal" variables), with higher values denoting more accurate models.

Supplementary Notes

Model and variable selection

Two model composition techniques are suitable to handle the large prevalence of postdocs without trainees, the *hurdle* and *zero-inflated* frameworks[2]. These models differ in how they account for researchers without trainees. The hurdle framework assumes that all independent researchers have at least one trainee, while the zero-inflated framework allows the existence of some independent researchers that have no trainee in the database. This latter scenario would correspond either to incomplete Academic Tree profiles or to researchers not involved in graduate/postdoctoral training. Furthermore, the proliferation rate may be modeled as a Poisson distribution or as a negative binomial distribution. The former assumes that count variance is directly proportional to mean count, while the latter relaxes this assumption and allows over-dispersion, at the cost of an extra free parameter.

To decide which of the four model architectures (hurdle or zero-inflated with Poisson or negative binomial distribution) and which predictors yielded the best fit to the data, we screened the performance of each architecture on all possible combinations of predictors. This was done by calculating parameter values for each model that maximized log-likelihood of predicted outcomes, using a 10-fold cross-validation scheme. Shapley values were then computed scoring the relative contribution of each variable to the overall model's performance. Negative binomial distributed count rates consistently outperformed count rates conforming to a Poisson distribution, indicating the presence of over-dispersion in rates for researchers with the highest proliferation rate. Zero-inflated models performed better than hurdle models, indicating that an assumption that all independent research faculty have at least one trainee is not consistent with this dataset (Supplementary Table 2). The best-fitting model overall was the zero-inflated negative binomial model. Cross-validated predictions for each input variable are shown in Fig. 12 in Supplementary Information. Given its superior performance, we focus on this mathematical model for the main results of the paper.

Supplementary References

1. Sue Doe Nihm. Polynomial law of sensation. *American Psychologist*, 31(11):808, 1976.

2. A Colin Cameron and Pravin K Trivedi. *Regression analysis of count data*, volume 53. Cambridge university press, 2013.
